# Uncovering sex patterns in BPSD among home-care patients: longitudinal findings from a population-based registry - digital dementia registry Bavaria

**DOI:** 10.3389/fpsyt.2026.1810510

**Published:** 2026-05-19

**Authors:** Lea Dütsch, Nikolas Dietzel, Anne Keefer, Lisa Laininger, Stefan Schwab, Thomas Ganslandt, Elmar Graessel, Peter L. Kolominsky-Rabas

**Affiliations:** 1 Department of Neurology, Uniklinikum Erlangen, Erlangen, Germany; 2 Interdisciplinary Center for Health Technology Assessment (HTA) and Public Health (IZPH), Friedrich-Alexander-Universität Erlangen-Nürnberg, Erlangen, Germany; 3 Medical Informatics, Friedrich-Alexander-Universität Erlangen-Nürnberg, Erlangen, Germany; 4 Center for Health Services Research, Uniklinikum Erlangen, Erlangen, Germany; 5 Department of Psychiatry and Psychotherapy, Friedrich-Alexander-Universität Erlangen-Nürnberg, Erlangen, Germany; 6 Psychiatric Health Services Research, Department of Psychiatry, Social Psychiatry, and Psychotherapy, Hannover Medical School, Hannover, Germany

**Keywords:** BPSD, dementia, MCI, registry, sex differences

## Abstract

**Background:**

Behavioral and psychological symptoms of dementia (BPSD) are core features of Alzheimer’s disease and related dementias. Previous research suggests possible sex differences in the prevalence and severity of BPSD; however, findings remain inconsistent. Moreover, most existing studies are cross-sectional and focus on institutionalized populations, while evidence from home-dwelling individuals is highly limited. As many people with dementia live at home for a substantial part of the disease trajectory, longitudinal analyses in this setting are essential.

**Methods:**

This study used data from the population-based, prospective Digital Dementia Registry Bavaria (digiDEM Bayern). A total of 368 home-dwelling individuals with mild cognitive impairment or mild to moderate dementia were included. BPSD were assessed at baseline and after 12 months using the Neuropsychiatric Inventory (NPI). Sex differences over time were examined using a mixed ANOVA, with age, educational level, and dementia severity included as covariates.

**Results:**

Overall, NPI scores showed a descriptive increase over the 12-month follow-up period. Men showed higher mean NPI scores than women at both measurement points. In unadjusted analyses, a significant main effect of time and a between-subjects effect of sex were observed. However, no significant interaction between sex and time was found. After adjusting for age, education, and dementia severity, neither time effects nor sex differences remained statistically significant.

**Conclusion:**

The findings suggest that sex differences in BPSD among home-dwelling individuals with dementia are not explained by sex alone. BPSD may be largely influenced by underlying demographic and clinical factors such as age, educational level, and dementia severity. This study highlights the importance of considering these covariates when examining BPSD and underscores the value of longitudinal, population-based research in home-care settings to better inform future dementia care strategies.

## 1 Introduction

Neuropsychiatric symptoms, also referred to as behavioral and psychological symptoms of dementia (BPSD), are considered core symptoms of Alzheimer’s disease and related dementias (1). BPSD occur in up to 90% of individuals over the course of dementia and typically include apathy, irritability, agitation, sleep and eating disturbances (2, 3). BPSD are associated with accelerated functional decline, increased caregiver burden, institutionalization, and higher mortality risk (4–9).

The prevalence and severity of BPSD are influenced by various factors, including age, the duration of the disease, severity of dementia, level of education and whether early-onset or late-onset dementia occurs (10–16).

Sex differences in dementia have been observed in terms of prevalence, symptoms, therapy, and treatment (17–19). Women are not only more frequently affected by dementia than men (20, 21) but also tend to present different BPSD symptoms (22), for example more depressive/anxious symptoms, whereas men more often show physical aggression, aberrant motor behavior, or apathy in some studies (23–25). With regard to sex differences in the severity of BPSD, some studies have found a wider and greater range in female participants compared to males (21, 26), whereas another study even reported a slightly higher NPI score for men (5). Despite these findings, sex-specific studies on BPSD remain scarce, and the heterogeneity of existing results highlights the need for further research (7, 23).

Given the progressive nature of dementia, it is crucial to capture the development and impact of BPSD over time. Therefore, a longitudinal approach is needed to adequately reflect these dynamic changes. To date, most studies investigating BPSD have relied on cross-sectional data and have primarily focused on individuals living in nursing homes, long-term care facilities, or memory clinics (3, 6, 12, 27, 28). However, given demographic changes and the increasing number of people with dementia living at home—at least during certain phases of the disease—there is an important need to further investigate to study BPSD in the home care setting.

The present study aims to investigate sex differences in the manifestation of BPSD among individuals with dementia living at home over a period of one year.

## 2 Materials and methods

This study is part of the ongoing project “Digital Dementia Registry Bavaria – digiDEM Bayern”. digiDEM Bayern is a population-based, multicenter, prospective, longitudinal registry study conducted in all administrative regions of Bavaria. The detailed methodology of the project is described elsewhere (29–31).

### 2.1 Study population

Participants are people with mild cognitive impairment (MCI) and people with mild or moderate dementia living in Bavaria, in the following referred to as people with cognitive impairment. To identify eligible participants, people have to undergo a screening based on the Mini-Mental State Examination (MMSE) and Montreal Cognitive Assessment (MoCA) prior to inclusion (32, 33).

### 2.2 Recruitment and data collection

Participants are recruited by specially trained research partners in all seven administrative regions of Bavaria, beginning in August 2020. Research partners are institutions that are specialized and have experience in the management and care of people with cognitive impairment and their family caregivers. Data collection is conducted through standardized face-to-face interviews using a web-based data entry system (29, 30, 34).

### 2.3 Neuropsychiatric assessment

Behavioral and psychological symptoms of dementia (BPSD) were measured using the Neuropsychiatric Inventory (NPI). It includes twelve different behavioural disturbances: delusions, hallucinations, dysphoria, anxiety, euphoria, agitation/aggression, apathy, disinhibition, irritability/lability, aberrant motor behaviour, behavioural disturbances as well as appetite and eating abnormalities (35, 36). According to Cummings et al. (35) a caregiver is someone, who assists the patient at least once per week reports on the patient’s behavioral and psychological symptoms. The caregiver is asked to rate the frequency of the mentioned symptoms included in the NPI on a scale from 1 to 4 (1 = occasionally, less than once per week; 4 = very frequently, once or more per day or continuously). In addition, the caregiver is asked to assess the severity of each symptom on a scale from 1 to 3 (1 = mild, 2 = moderate, 3 = severe). The use of NPI, as a validated instrument, allows our study to be compared to former studies (12, 21, 37).

### 2.4 Sociodemographic factors

Sociodemographic data were collected at baseline. We included age, dementia severity, and years of education as covariates, as these variables are consistently associated with the occurrence and severity of BPSD. Adjusting for these potential confounders allows a more accurate estimation of sex differences in BPSD (3, 4, 6, 15, 38–41). Dementia severity was assessed using the Mini-Mental State Examination (MMSE) at baseline and included as a covariate in all adjusted analyses to account for differences in cognitive status at study entry.

### 2.5 Ethical considerations and data security issues

digiDEM Bayern obtained ethical approval from the ethics committee of the Medical Faculty of the Friedrich-Alexander-Universität Erlangen-Nürnberg (application number: 253_20 B). Informed consent from the participants or their authorized representative is acquired before screening and study inclusion.

The project collects and stores all personal data separately from the registry data on different stand-alone systems to ensure data protection (30). The data protection concept was approved by the local Data Protection Officer of the Friedrich-Alexander-Universität Erlangen-Nürnberg and reviewed by the Bavarian Data Protection Commissioner. Participation is voluntary, and participants are not compensated (29).

### 2.6 Statistical analysis

A mixed ANOVA was performed with the prior described variables and covariates. Analyses were conducted using a complete-case approach, including only participants with available data at both baseline and 12-month follow-up. Assumptions underlying the mixed ANOVA were assessed prior to analysis. Normality of residuals was examined using graphical methods and appropriate statistical tests and was considered acceptable after transformation. Homogeneity of variance was confirmed using Levene’s test.

Data analysis was conducted using IBM Statistical Package for the Social Sciences (SPSS) version 28.

## 3 Results

### 3.1 Participants

**Figure 1:**
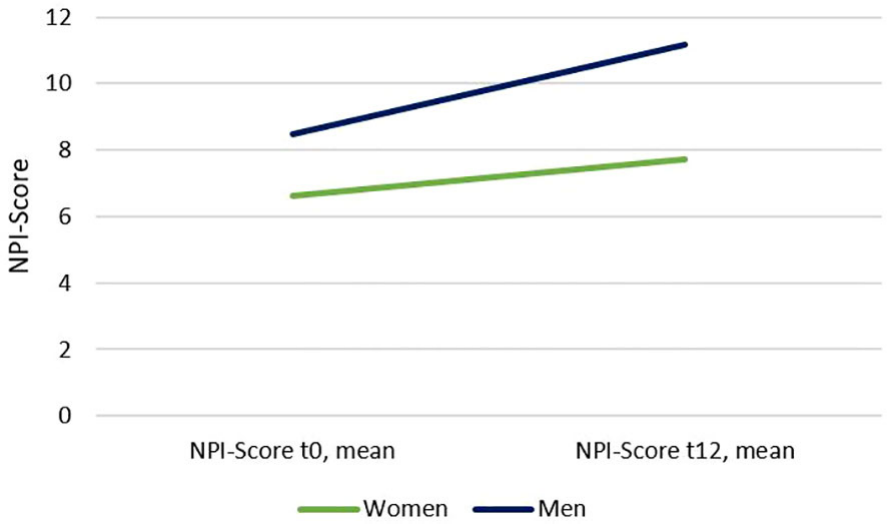
Descriptive NPI-progression by gender.

A total of 368 participants were included in this study, 174 (47.28%) with MCI and 194 (52.72%) with mild or moderate dementia. 203 (55.2%) of all participants were female. The mean age at baseline was 80 years (SD = 7.73). Overall, the participants spent an average of 9.3 (SD = 1.66) years in school. Mean of years spent in school was higher by men than by women (9.85, SD = 1.93 compared to 8.9, SD = 1.26). At baseline, the overall mean MMSE score was at 22.1 (SD = 3.55) and the mean of the MMSE after 12 months was 20.6 (SD = 5.1). The overall mean NPI Score at baseline was at 7.47 (SD = 10.75) and has increased to 9.27 (SD = 13.13) after 12 months. Mean NPI score from women raised from 6.64 (SD = 9.67) at baseline to 7.71 (SD = 11.67) (**Figure 1**). Mean NPI score from men raised from 8.5 (SD = 11.9) at baseline to 11.19 (SD = 14.53) (Graph 1). Descriptive statistics of the sample can be seen in **Table 1**.

**Table 1:** The demographic characters of recruited subjects by sex.

Mean (SD)/no. (%)						
Sample characteristics	Total (n=368)	MCI (n=174)	Dementia (n=194)	Women (n=203)	Men (n=165)	p-value^a^
Females, in %		89 (51.15)	114 (58.76)			
Age, mean, years	80.2 (7.73)	79.17 (7.56)	81.06 (7.78)	81.22 (7.26)	78.9 (8.1)	0.004
Years of formal Education, mean	9.33 (1.66)	9.4 (1.69)	9.26 (1.64)	8.9 (1.26)	9.85 (1.93)	<0.001
MMSE t0, mean, score	22.1 (3.55)	25.13 (1.96)	19.39 (2.19)	21.9 (3.43)	22.35 (3.68)	0.236
MMSE t12, mean, score	20.6 (5.07)	23.61 (3.8)	17.90 (4.54)	20.6 (4.57)	20.61 (5.63)	0.993
NPI Score t0, mean, score	7.47 (10.75)	6.31 (8.73)	8.51 (12.21)	6.64 (9.67)	8.5 (11.9)	0.053
NPI Score t12, mean, score	9.27 (13.13)	8.13 (11.11)	10.30 (14.66)	7.71 (11.67)	11.19 (14.53)	0.013

MMSE, Mini-Mental State Examination; NPI, Neuropsychiatric Inventory. ^a^T-test for continuous variables.

### 3.2 Mixed ANOVA

To further examine the contribution of covariates, stepwise models were conducted.

In the unadjusted model, no significant interaction between sex and time was observed (F (1, 366) = 2.601, p = .108), while both the within-subjects factor (time) (F (1, 366) = 5.100, p = .025) and the between-subjects factor (sex) (F (1, 366) = 7.026, p = .008) showed small but significant effects.

After adjustment for age and education, these effects were no longer statistically significant. The inclusion of MMSE as an additional covariate did not change this pattern, as no significant effects of sex, time, or their interaction were observed in the fully adjusted model.

### 3.3 Stratified analysis

Stratified analyses were conducted separately for participants with MCI and those with dementia. Overall, the stratified results were consistent with the main analyses, as initially observed sex differences were no longer statistically significant after adjustment for covariates.

In the unadjusted analyses, a significant main effect of sex was observed in the MCI group (F (1, 172) = 3.945, p = .049, partial η² = .022), indicating higher NPI scores in men compared to women, while no significant effects were observed in the dementia group (all p >.05).

In the MCI group, after adjustment for age and education, no significant effect of sex was observed (p = .129). After adjustment for age and education, as well as in the fully adjusted models including MMSE, no significant effects of sex, time, or their interaction were observed in either group.

Age remained a significant factor in the between-subjects analysis (F (1, 189) = 8.789, p = .003, partial η² = .044).

## 4 Discussion

To our knowledge, this is the first study investigating sex differences in the manifestation of BPSD among home-dwelling individuals with dementia, based on long-term data (3) from a population-based registry.

Our analysis showed that women were older, had fewer years of education, and exhibited lower NPI- and MMSE-scores at baseline and after 12 months compared to men. These descriptive findings are consistent with a study from Taiwan, which also reported higher education levels and higher MMSE scores in men (21).

We could not find any significant interactions between sex as group factor and time. Previous research indicated that education (15) and dementia severity (12–14) contribute to the presentation and prevalence of BPSD. After including the covariates age, severity of dementia and education, no significant effects were found. This suggests that sex differences in BPSD may be partly explained by these factors.

Exploratory stratified analyses were conducted to further examine potential differences between individuals with MCI and those with dementia.

In the MCI group, small sex differences observed in unadjusted analyses were no longer statistically significant after adjustment for age, education, and dementia severity. This suggests that the initially observed differences may be explained by underlying demographic and clinical factors rather than sex alone.

In contrast, no significant effects of sex, time, or their interaction were observed in the dementia group after adjustment, while age remained a relevant factor in the between-subjects analysis.

Across all stratified models, findings were consistent in showing that the initially observed sex differences were attenuated after accounting for demographic and clinical variables.

Overall, these results support the assumption that demographic and clinical factors, particularly age and disease context, may play a more important role than sex alone in explaining differences in BPSD. However, as these analyses were exploratory and effects were small and not consistently observed across models, they should be interpreted with caution. Alternative explanations such as residual confounding, measurement limitations, or limited statistical power cannot be excluded.

A major strength of this study is its focus on people with dementia living at home, whereas other studies on sex differences in BPSD have focused on individuals in nursing homes, long-term facilities or memory clinics (12, 27, 28, 42). Prior research also often included patients with more advanced dementia. As dementia severity is known to influence BPSD and NPI scores (12–14, 43), individuals living at home, who typically have MCI or mild to moderate dementia, tend to show lower symptom burden.

This explains the lower NPI scores observed in our study compared to other studies investigating sex differences in BPSD, as they included patients with severe dementia recruited from institutional settings (12, 13, 15, 21, 28). By focusing on home-dwelling individuals, this study contributes to addressing a gap in the existing literature.

Several limitations should be considered. Participants were recruited across all regions of Bavaria, but recruitment strategies may have varied, which could introduce selection bias and limit generalizability. The follow-up period of 12 months is relatively short; however, in home-dwelling populations, longer follow-up periods are difficult to achieve, as disease progression increases the likelihood of institutionalization and dropout. While longer follow-up periods might provide additional insights into the progression of BPSD and the emergence of group differences over time, the present design may not fully capture these longer-term developments. Dementia severity was assessed solely using MMSE scores. Although the MMSE is a widely used and standardized screening tool, it may not fully reflect the multidimensional nature of dementia severity. At the same time, the population-based design of digiDEM Bayern, including participants from rural and underserved regions with limited access to specialized diagnostics, requires the use of feasible and scalable assessment methods. In addition, analyses were based on the NPI total score, which captures overall symptom burden but may obscure domain-specific patterns. Social and other non-pharmacological interventions, as well as social factors such as living situation and family context, were not included in the present analysis, although they may influence BPSD. Furthermore, the distribution of NPI scores showed some degree of skewness, which may affect the assumptions of parametric analyses; however, mixed ANOVA is generally considered robust to such deviations in larger samples. Finally, comorbid chronic and mental health conditions were not considered and may also influence the presentation of BPSD and potential sex differences.

## 5 Conclusion

Our study investigated sex differences in BPSD in a home-care setting based on population-based data. Our models showed no significant results and suggest that differences may not be attributable to sex alone but rather to underlying demographic factors such as age, dementia severity and education level. Our findings highlight and support the importance of considering sociodemographic and clinical factors when examining BPSD.

As the number of people living with dementia continues to rise globally, healthcare systems will face increasing financial and structural challenges in the coming decades (46, 20a). These challenges are further amplified by the worldwide shortage of healthcare professionals and the unequal distribution of nursing staff (47, 48). The group of home-dwelling people with dementia is often overlooked, even though most individuals with dementia spend the majority of their course of disease in their own homes (44, 45). Mjørud et al. (2020) found that four years post-diagnosis, half of the study participants still lived in their private households. Similarly, a recent systematic review reported that only one third of the remaining life expectancy is spent in nursing homes (45).

In the context of global demographic change, this is an important finding as more individuals require long-term care and progressively intensive support, the demand for sustainable care structures increases. Long-term care costs are already high and are expected to rise in all OECD countries, placing a burden not only on individuals but also on public systems (49, 50). Considering the high costs of institutional care and the simultaneous shortage of qualified staff, home-based care emerges as a particularly relevant and future-oriented form of support.

Strengthening outpatient and community-based services is therefore essential. Expanding these structures can relieve families, reduce pressure on the healthcare system, and ensure that people with dementia can live at home for as long as possible, which is the preferred place to stay and often also the place where they experience the highest quality of life.

## Data Availability

The raw data supporting the conclusions of this article will be made available by the authors, without undue reservation.
